# The visible and hidden blood loss of Asia proximal femoral nail anti-rotation and dynamic hip screw in the treatment of intertrochanteric fractures of elderly high- risk patients: a retrospective comparative study with a minimum 3 years of follow-up

**DOI:** 10.1186/s12891-016-1143-3

**Published:** 2016-07-11

**Authors:** Weiguang Yu, Xinchao Zhang, Rongbo Wu, Xingfei Zhu, Jun Hu, Yinfeng Xu, Jianhua Yi, Yunjiang Liu

**Affiliations:** Department of Orthopedics, The First Affiliated Hospital of Sun Yat-sen University, Huangpu East Road No. 183, Huangpu District, Guangzhou City, Guangdong Province 510700 China; Department of Orthopaedics, Jinshan Hospital, Fudan University, Longhang Road No. 1508, Jinshan District, Shanghai City, 201508 China; Department of Orthopedics, Tongji Hospital, Tongji University School of Medicine, Xincun Road No. 389, Shanghai City, 200065 China

**Keywords:** Intertrochanteric fractures, Dynamic hip screw, Asia proximal femoral nail anti-rotation, Blood loss

## Abstract

**Background:**

The purpose of this study was to evaluate whether PFNA-II (Asia proximal femoral nail anti-rotation) and DHS (dynamic hip screw) carry substantial post-operative hidden blood loss and to compare PFNA-II with DHS in terms of post-operative hidden blood loss in elderly high-risk patients with intertrochanteric femur fractures(IFFs).

**Methods:**

The clinical data from Jan 2005 to Apr 2015 of 186 patients with PFNA-II and 177 patients with DHS were analyzed retrospectively. Indexes including pre- and post-operative blood routine, intra- and post-operative blood loss and blood transfusion situation were analyzed. The situation of perioperative blood loss (visible and hidden) was assessed.

**Results:**

The intra-operative blood loss in the PFNA-II group was 34.7 ± 2.5 ml, the post-operative visible blood loss was 54.7 ± 2.5 ml, and the hidden blood loss was 277.2 ± 7.6 ml. In the DHS group, the intra-operative blood loss was 102.0 ± 7.0 ml, the post-operative visible blood loss was 78.8 ± 4.7 ml, and the hidden blood loss was 139.3 ± 9.6 ml. The intra-operative blood loss and the post-operative visible blood loss in the PFNA-II group were significantly less than in the DHS group (*p* < 0.01). However, the post-operative hidden blood loss and the total blood loss in the PFNA-II group were larger than in the DHS group (*p* < 0.01).

**Conclusion:**

This study demonstrated that with PFNA-II and DHS, much post-operative hidden blood loss exists in the treatment of intertrochanteric fractures in elderly high-risk patients and DHS is more favourable than PFNA-II in terms of post-operative hidden blood loss.

## Background

With the rapid increase in the elderly population, intertrochanteric femur fractures(IFFs) have become a serious health issue because they are common, morbid, costly and associated with injury [1–3]. To remedy this, two effective treatment methods, conservative treatment and surgical intervention. There is a high rate of the complication of prolonged immobilization, pneumonia, decubitus ulcers, joint contractures, and thromboembolism in the conservative treatment, which often contributes to high mortality [4–7]. The increased incidence of shortening and introversive deformity results in dysfunction. Hence, the treatment is almost always surgical [8, 9]. The most common options are either the dynamic hip screw (DHS) or Asia proximal femoral nail anti-rotation (PFNA-II) [10]. Current evidence-based clinical research, however, has revealed that DHS may cause more intra- and post-operative complications [8, 11–14]. PFNA-II can minimize the risk of these implant-related complications and provide angular and rotational stability. This is especially important in osteoporotic bone and unstable IFFs of elderly high-risk patients, allowing early mobilization and weight bearing on the affected limbs [15–18]. The literature reports that the biomechanical stability of PFNA-II is superior to that of DHS [17, 19]. However, there is no definitive conclusion thus far on which fixation method is optimal for elderly high-risk patients with IFFs in reducing hidden blood loss and improving prognosis.

Clinically, post-operative hidden blood loss is often ignored in elderly high-risk patients, due to a relatively simpler surgical procedure, shorter operative time and less intra-operative visible blood loss [20]. Consequently, critical patients are not dealt with in a timely manner or experience other complications.

Recently, a large number of studies assessing blood loss in the two fixed methods of IFFs have been performed [21–23]. However, these studies have important limitations in sample size, data processing, and methodology quality and fail to draw a definitive conclusion on whether PFNA-II or DHS result in substantial post-operative hidden blood loss. Thus, to provide strong support for a clinical decision, the clinical case data of 363 patients with IFFs accepting the treatment with PFNA-II or DHS were analyzed retrospectively to evaluate blood loss. Additionally, we also propose some measures to reduce mortality.

## Methods

### General data

This study was reviewed and approved by the Medical Ethical Committee (the First Affiliated Hospital, Sun Yat-sen University), and an exemption from informed consent was obtained from our responsible Investigational Ethical Review Board.

In this study, 1363 confirmed patients with an IFF treated using the PFNA-II or DHS in our Department of Orthopaedics, the First Affiliated Hospital of Sun Yat-sen University, from January 2005 to April 2015, were enrolled consecutively. There were 19 deaths among these patients (7 deaths (0.5 %) due to pulmonary embolism within 30 days after surgery, 9 deaths (0.7 %) due to heart arrest and 3 deaths (0.2 %) secondary to cerebral haemorrhage before surgery). The patient data were fully anonymized and de-identified prior to access by the researchers. Our inclusion criteria were as follows: ages ranging 65–95 years, good cognitive function, IFFs (AO/OTA Type 3.1A1, 2, 3), ability to walk independently without aids before fracture, surgery performed on an average of 3 days (range, 1–6 days) after admission, and undergoing a DHS or PFNA-II fixation (DHS: standard screw/blade; Synthes, West Chester, PA, USA; PFNA-II: a solid titanium nail, 200/240 mm in length, 11 or 12 mm in diameter, 125° or 130° in collodiaphyseal angle (CCD), Smith & Nephew, Memphis, Tennessee). Our exclusion criteria were as follows: pathological fractures or the presence of metastatic disease, poly-trauma, severe osteoarthritis, chemotherapy, the inability to walk before injury, an American Society of Anaesthesiologists (ASA) score of V, hematologic diseases, laboratory signs of bleeding disorders, diseases of erythrocyte injury, consumption of non-steroidal anti-inflammatory drugs or antithrombotic drugs, antiplatelet agents taken within one month before surgery, chronic or acute liver diseases, organ failure diseases, substantial blood loss (>1.0 L), deaths, infectious diseases (e.g., acquired immune deficiency syndrome), diabetes, hypertension, or mental illness (Table 1).

**Table 1:** Major exclusion criteria

	PFNA-II^b^	DHS^c^
Grand total	285	304
Hypertension (n)	51	45
Diabetes (n)	52	53
Hypertension and diabetes (n)	12	13
Rheumatoid arthritis (n)	8	12
Steroid consumption (n)	11	10
Kidney failure (n)	6	4
Hemodialysis (n)	5	11
Delirium and agitation (n)	2	1
Liver cirrhosis (n)	15	18
Organ transplant (n)	4	3
Oxygen dependence (n)	3	6
Hemodynamic instability^a^ (n)	1	2
Concomitant use of heparin (n)	5	3
Multiple organ failure (n)	6	3
Heart failure (n)	3	9
Deaths (n)	12	7
Mental illness(n)	1	2
More blood loss (>1.0 L) (n)	2	6
Consumption of non-steroidal anti-inflammatory drugs (n)	8	6
Diseases of erythrocyte injury (n)	5	1
Laboratory signs of bleeding disorders (n)	4	3
Hematologic diseases (n)	3	7
ASA score of V (n)	4	11
Inability to walk before injury (n)	5	8
Chemotherapy (n)	10	11
Poly-trauma (n)	13	22
Pathological fractures (n)	10	7
Multiple above mentioned indicators (n)	24	20

^a^Requirement for multiple pressors or inability to maintain mean arterial pressure with single-pressor support. PFNA-II: Asia proximal femoral nail anti-rotation; *DHS* dynamic hip screw. ^b^Smith & Nephew, Memphis, Tennessee; ^c^Synthes, West Chester, PA, USA

**Fig. 1:**
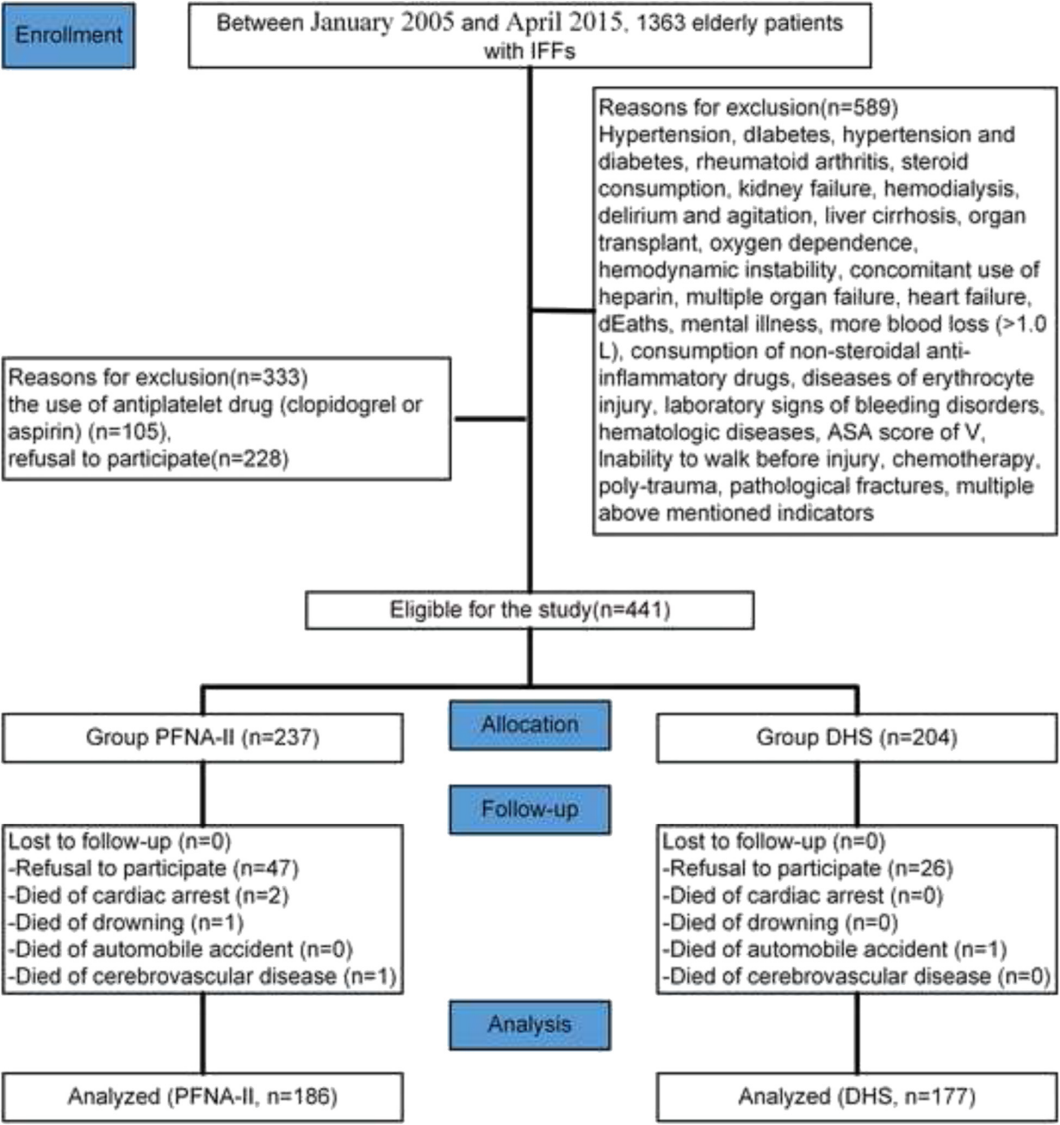
Flow diagram demonstrating methods for identification of studies to assess the treatment of intertrochanteric femur fractures in the elderly using either the Asia proximal femoral nail anti-rotation (PFNA-II) or the dynamic hip screw (DHS) systems, and reasons for exclusion

Based on these criteria, 922 patients were excluded. In those patients, 105 patients were excluded because of the use of antiplatelet drug (clopidogrel or aspirin). Another 228 patients refused to participate, leaving 441 patients eligible for the study. During the follow-up, 73 patients refused to continue participating in the study. Two patients died from cardiac arrest, and 3 patients died of drowning, cerebrovascular disease, and an automobile accident. Consequently, 363 patients (375 primary operations, PFNA-II, *n* = 186; DHS, *n* = 177) were involved in the final evaluation (Table 2, Fig. 1). Osteoporosis was defined as bone mineral density(BMD)T-score value ≤ −2.5 at the femoral neck.

**Table 2:** Patient details in this study

	PFNA-II^b^		DHS^c^		*p*-value
Gender	Men	Women	Men	Women	
Total limbs (n)	194		181		
	81	113	80	101	0.632^#^
Total patients (n)	186		177		
	75	111	78	99	0.470^#^
Age range (y)	66–82	67–92	64–82	63–95	0.273^#^
Mean age (y)	76.4	79.6	74.6	80.3	
Left side (n)	52	67	50	42	0.125^#^
Right side (n)	29	46	30	59	0.510^#^
BMD	−3.73 ± 1.21		−3.98 ± 1.49		0.086^#^
Type of fracture					0.385^#^
Type-A1 fracture (n)	33	56	40	40	
Type-A2 fracture (n)	24	24	18	38	
Type-A3 fracture (n)	23	33	22	23	
Length of hospital stay (d)	9.91 ± 3.47		10.40 ± 2.96		0.151^#^
Post-op HHS^a^	85.63 ± 4.13		82.59 ± 5.01		0.000*
Length of follow-up (mos)	39.58 ± 1.67		39.77 ± 1.41		0.234^#^

*Statistically significant values, ^#^No statistically significant values, ^a^At final follow-up

*PFNA-II* Asia proximal femoral nail anti-rotation-Asia; *DHS* dynamic hip screw; *HHS* Harris hip score; *BMD* Bone mineral density. ^b^Smith & Nephew, Memphis, Tennessee; ^c^Synthes, West Chester, PA, USA

### Data collection

Recorded clinical data of patients included the following: age, gender, body weight, height, and pre- and post-operative routine blood parameters (hematocrit, HCT; hemoglobin, Hb). The mean and SD of hemoglobin level changes are shown in Table 3.

**Table 3:** Pre-and post-operative change in hemoglobin levels (x ± s)

	PFNA-II^a^	DHS^b^	*p*-value
Preoperative hemoglobin level (g/L)	125.07 ± 1.60	123.40 ± 1.79	0.098^#^
Preoperative Hct (%)	32.64 ± 2.59	32.13 ± 2.59	0.837^#^
Postoperative hemoglobin level (g/L)	103.59 ± 2.01	101.39 ± 2.31	0.016^#^
Postoperative Hct (%)	27.05 ± 0.53	25.92 ± 0.61	0.009*
Hemoglobin level loss (g/L)	21.47 ± 2.40	22.01 ± 2.98	0.001*
PT(s)	10.0 ± 0.6	10.0 ± 0.59	0.195^#^
APTT(s)	26.2 ± 1.7	26.0 ± 1.8	0.337^#^
INR	1.0 ± 0.1	1.0 ± 0.1	0.351^#^

*Statistically significant values, ^#^No statistically significant values

*PFNA-II* Asia proximal femoral nail anti-rotation-Asia; *DHS* dynamic hip screw; *Hb* hemoglobin; *PT* prothrombin time; *APTT* activated partial thromboplastin time; *INR* international normalized ratio; *Hct* haematocrit. ^a^Smith & Nephew, Memphis, Tennessee; ^b^Synthes, West Chester, PA, USA

### Intra-operative and post-operative treatment

Each patient was given a single dose of the antibiotic teicoplanin (Aventis Pharma, Kings Hill, United Kingdom) 30 min before surgery. The surgery was performed under the direct supervision of senior surgeons. Patients were operated on an extension table and positioned in the same decubitus. Under general anaesthesia, each patient was treated with a DHS or PFNA-II fixation device. All operations were performed in the same institution by experienced orthopaedic surgeons (WY, YL, JY, YX, and JH). The technique followed standard protocols and was identical to that described by Zhong et al. [8] for PFNA and Jung et al. [9] for DHS. The length of incision in each group is similar to recommended length (standard incisions). The operation time lasted from the starting incision to the finishing suture of the incision. Intraoperative blood loss was measured gravimetrically and added to the blood loss measured in the suction bottles. The mass of haemoglobin lost in suction was calculated as the product of the suction volume and suction haemoglobin concentration [Vol_suction_*HbConc_suction_ = HbMass_suction_]. The total volume of blood lost in swabs was calculated as the sum of the changes in weight of all swabs. The mass of haemoglobin lost in swabs was calculated using the assumption that the haemoglobin concentration in the swabs would be similar to that of the suction [Vol_swabs_ *HbConc_suction_ = HbMass_swabs_]. The wound drainage was that obtained and measured from the wound suction drain following surgery. Twenty-four hours later, the drainage tube was removed according to the drainage condition, and the total drainage of post-operation (visible blood loss) was recorded. All patients had their blood routine test performed at 7:00 AM of the pre-operative day and on the second post-operative day, and the appropriate treatment was carried out according to the conditions of patients. Continuous passive motion (CPM) was started on the 2nd-3rd post-operative day.

### Calculation method of hidden blood loss

Red blood cell amount, perioperative blood loss and hidden blood loss were calculated using the Gross formula [24]. The details of the calculation method are as follows [25–27]:

$$ \mathrm{Patient}'\mathrm{s}\ \mathrm{total}\ \mathrm{blood}\ \mathrm{volume}\ \left(\mathrm{P}\mathrm{B}\mathrm{V}\right)\left(\mathrm{in}\ \mathrm{liters}\right) = \mathrm{k}1*\mathrm{h}3 + \mathrm{K}2*\mathrm{w} + \mathrm{K}3 $$

where h = height in metres, and w = weight in kg

Men: K1 = 0.3669, K2 = 0.03219, K3 = 0.6041,

Women: K1 = 0.3561, K2 = 0.03308, K3 = 0.1833.

$$ \mathrm{Total}\ \mathrm{blood}\ \mathrm{loss}\left(\mathrm{in}\ \mathrm{liters}\right) = \mathrm{P}\mathrm{B}\mathrm{V}*\left(\mathrm{Hctpre}-\mathrm{Hctpost}\right)*\mathrm{Hctave}. $$

Hctpre: the initial pre-operative Hct,

Hctpost: the Hct for the second or third post-operative day,

Hctave: the average of Hctpre and Hctpost,

$$ \mathrm{Hidden}\ \mathrm{blood}\ \mathrm{loss} = \mathrm{calculated}\ \mathrm{total}\ \mathrm{blood}\ \mathrm{loss} + \mathrm{blood}\ \mathrm{infused}-\mathrm{measured}\ \mathrm{blood}\ \mathrm{loss}. $$

### Statistical analysis

All statistical analyses were performed using SPSS Statistics version 22. All continuous data were expressed as the mean ± standard deviation (SD). Quantitative variables were analyzed using Student’s *t*-test and categorical variables were analyzed by the *χ*2 test or Fisher’s exact test where appropriate. All tests were two-tailed. The level of significance was set at *p* < 0.01 for all statistical analyses.

## Results

**Fig. 2:**
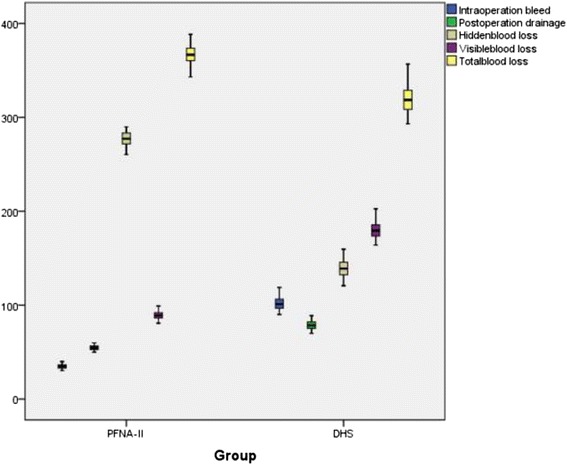
Box plots showing the characteristics of its statistical distribution. DHS, dynamic hip screw; PFNA-II, Asia proximal femoral nail anti-rotation

**Fig. 3:**
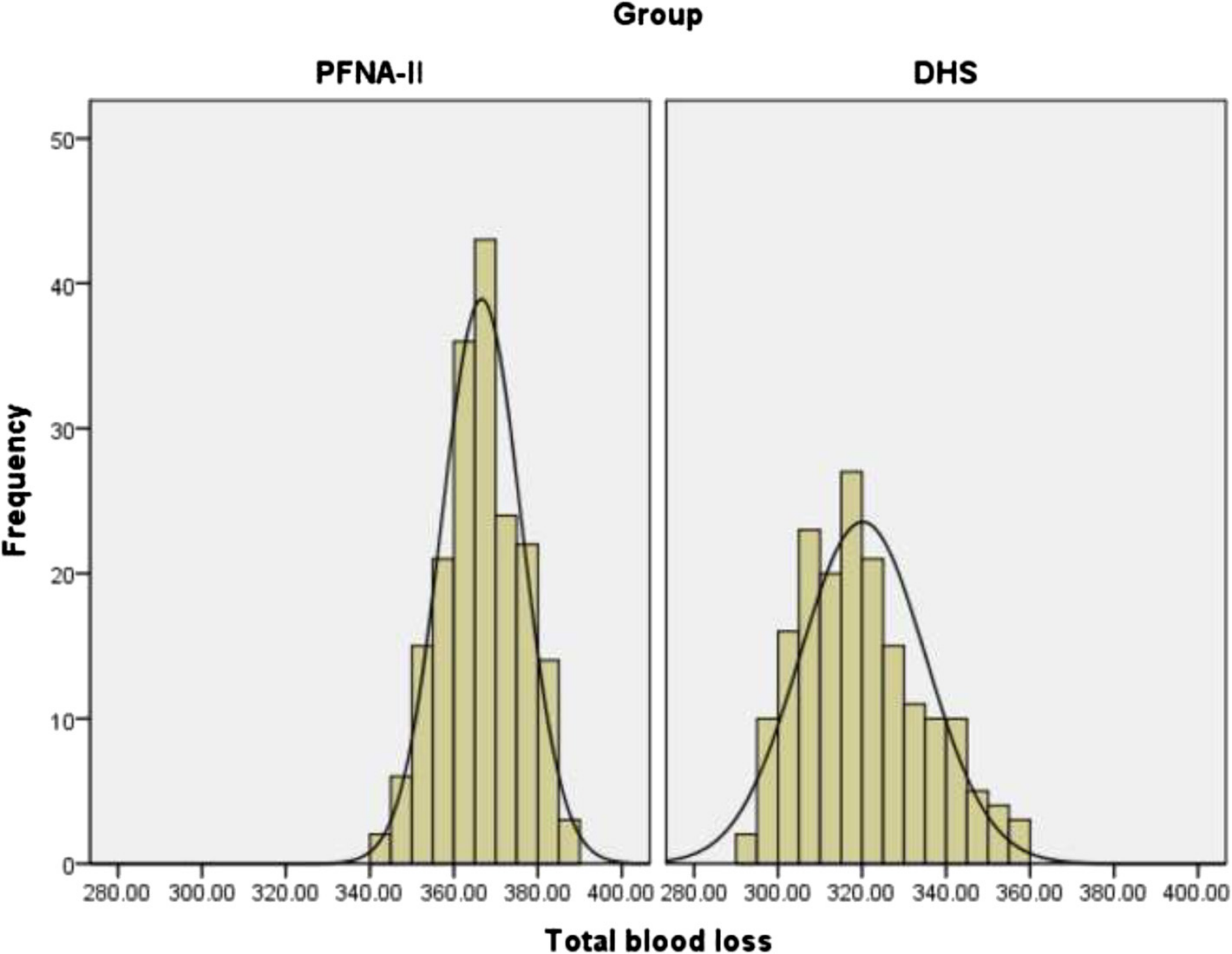
Frequency distribution of the total blood loss measurements. Its concentration is shown as the obviously centralized distribution in two groups, and in Group PFNA-II, most of the values are approximately 368 ml, in Group DHS, approximately 320 ml.DHS, dynamic hip screw; PFNA-II, Asia-proximal femoral nail anti-rotation

**Fig. 4:**
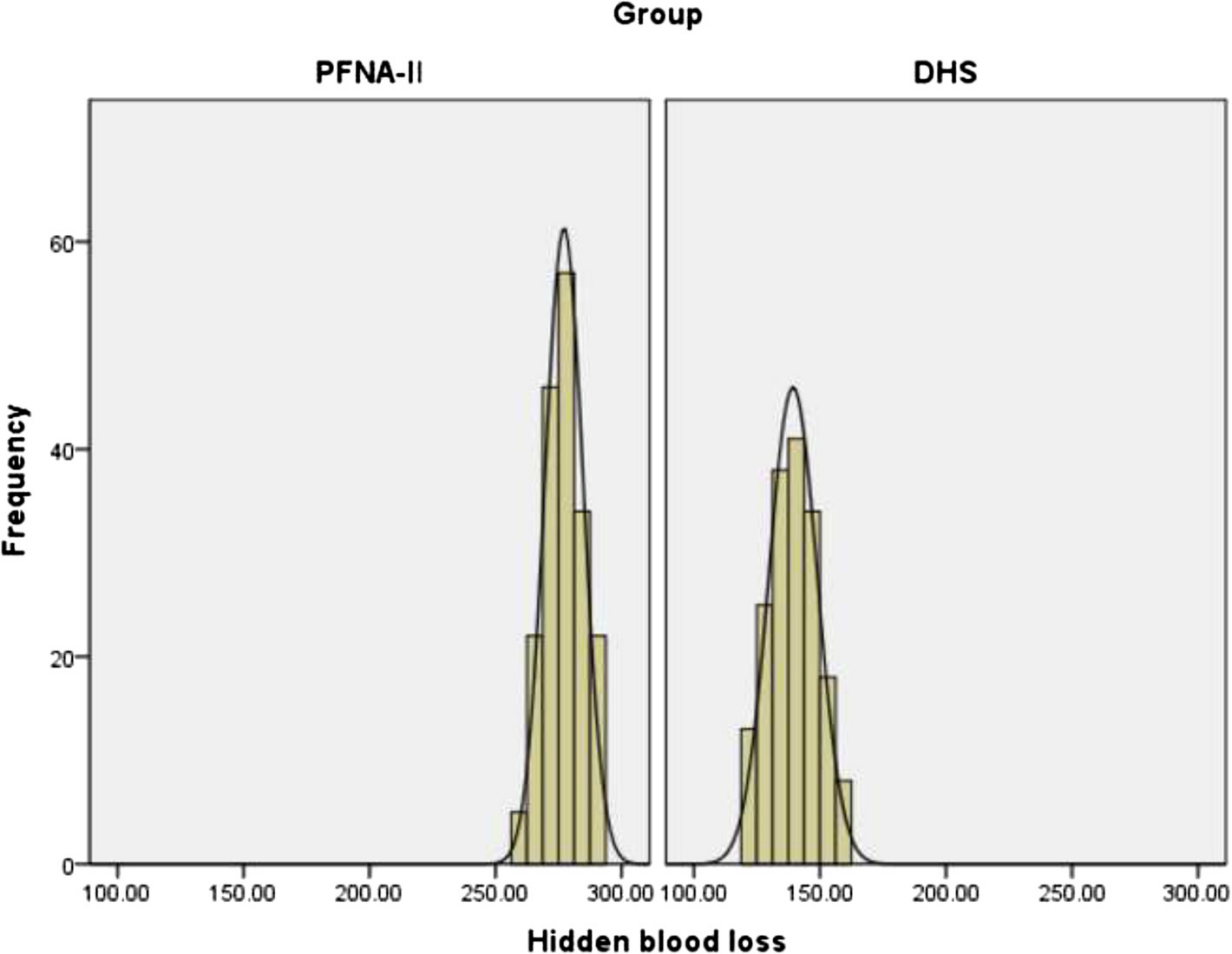
Frequency distribution of the hidden blood loss measurements. Its Concentration is shown as the obviously centralized distribution in two groups, and in Group PFNA-II, most of the values are approximately 277 ml, in Group DHS, approximately 139 ml. DHS, dynamic hip screw; PFNA-II, Asia proximal femoral nail anti-rotation

**Fig. 5:**
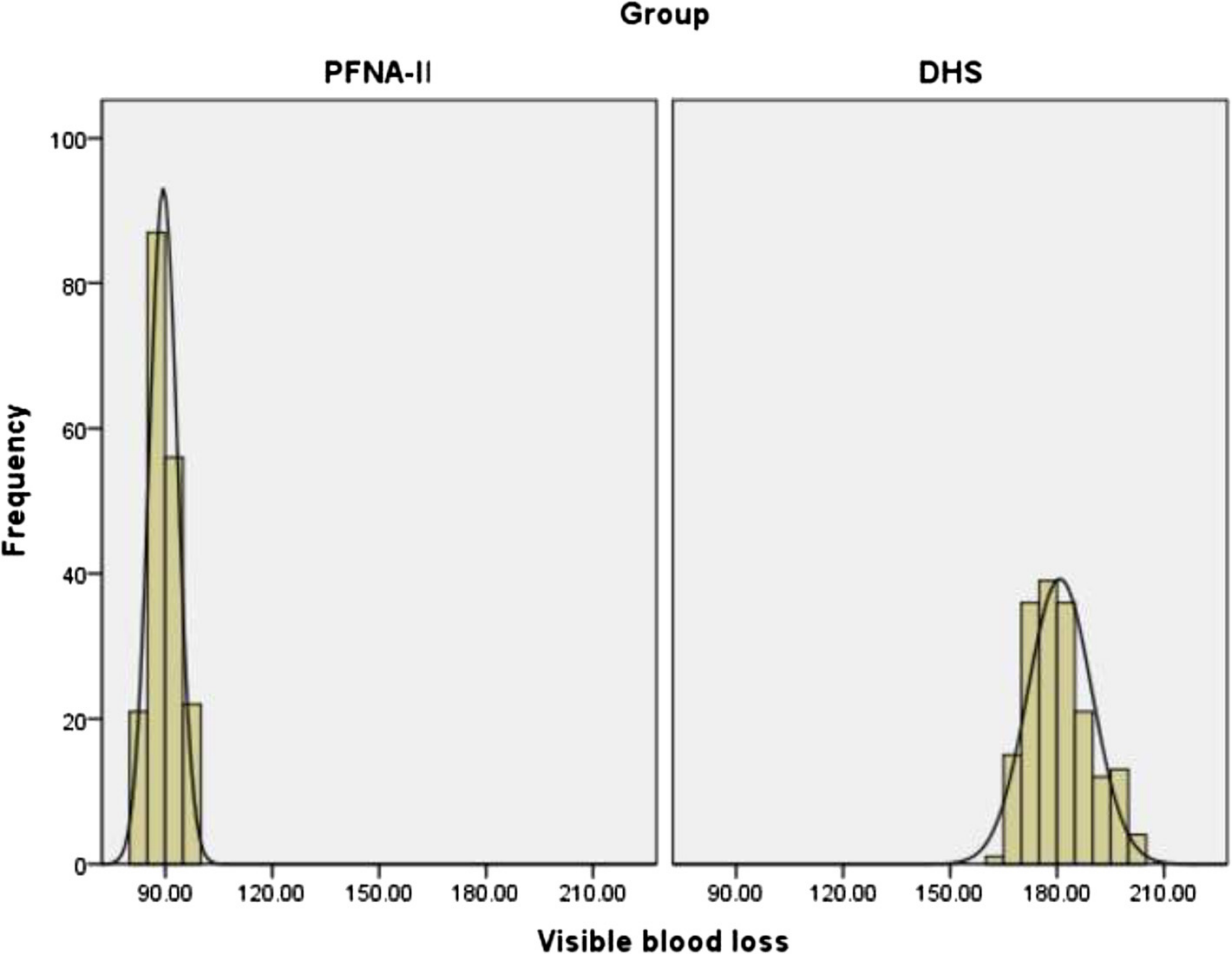
Frequency distribution of the visible blood loss measurements. Its concentration is shown as the obviously centralized distribution in two groups, and in Group, most of the values are approximately 89 ml, in Group DHS, approximately 180 ml.DHS, dynamic hip screw; PFNA-II, Asia proximal femoral nail anti-rotation

**Fig. 6:**
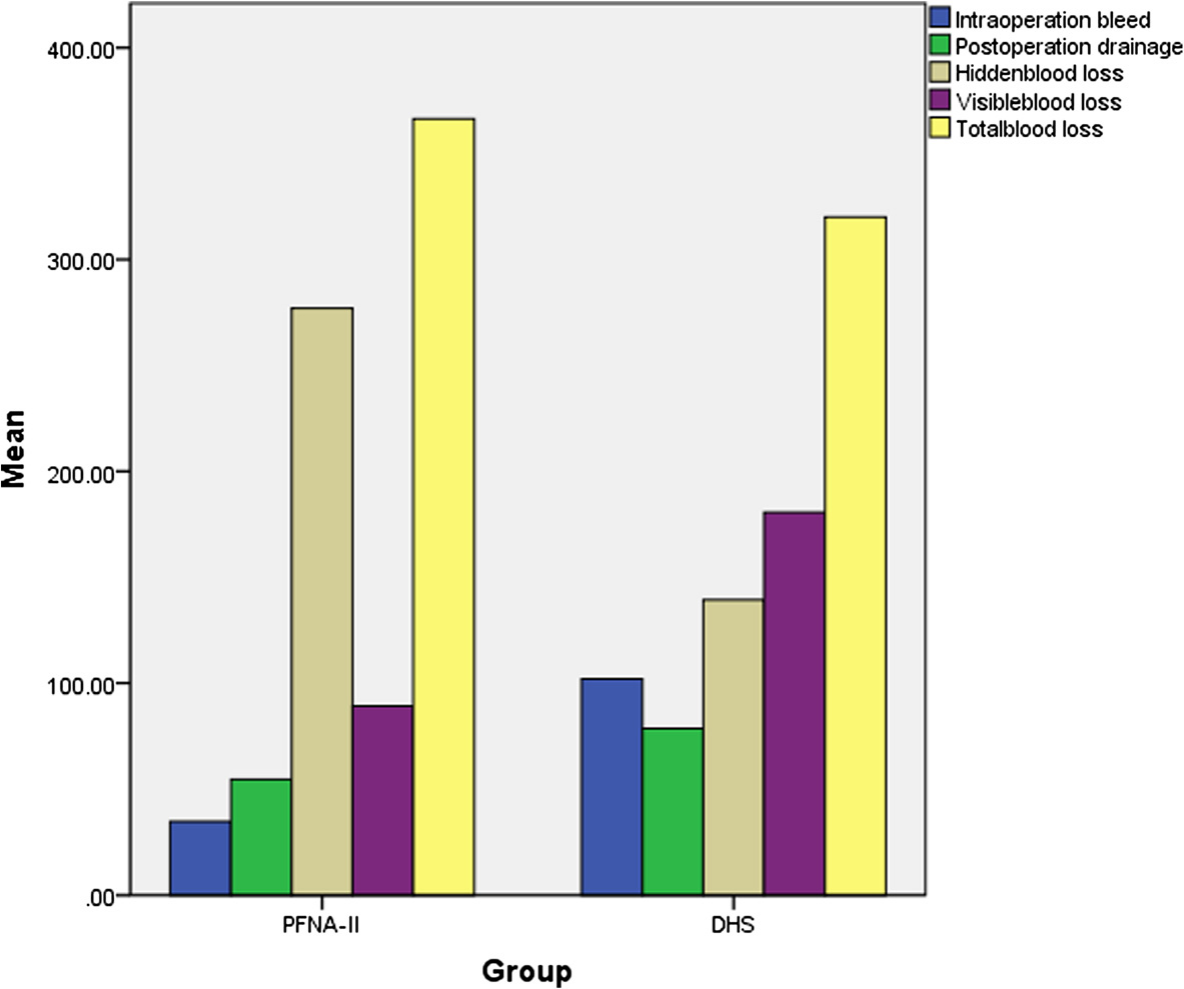
Variable mean differences between two groups: in Group DHS, IOB and POD are larger than that in Group PFNA-II, HBL and TBL are less than that in Group PFNA-II. DHS, dynamic hip screw; PFNA-II, Asia proximal femoral nail anti-rotation; IOB, Intra-operation bleed; POD, Post-operation drainage; HBL, Hidden blood loss; TBL, Total blood loss

The details of the hidden and visible blood loss with PFNA-II and DHS are shown in Table 4. Box plots show the distribution characteristic of measured data (Fig. 2). The frequency distribution of the total blood loss measurements was that its concentration appeared as a clearly centralized distribution in both groups: in Group PFNA-II, most of the values are approximately 368 ml, whereas in Group DHS, the values were approximately 320 ml (Fig. 3). As for hidden blood loss measurements, the concentration also appeared as a clearly centralized distribution in both groups: in Group PFNA-II, most of the values were approximately 277 ml, in Group DHS, approximately 139 ml (Fig. 4). A characteristics of the statistical distribution of visible blood loss revealed that in Group PFNA-II, most of the values were approximately 89 ml, whereas in Group DHS, the values were approximately 180 ml (Fig. 5). Variable mean differences between two groups are shown in Fig. 6. In Group PFNA-II, the mean operation time was 35.5 ± 1.4 min. The mean intra-operation blood loss was 34.7 ± 2.5 ml, without blood transfusion during operation. Due to a significant decrease in blood red protein (<8 g/L) on the 2nd-4th post-operative days, 36 patients were provided with a blood transfusion, which averaged 300 ± 15 ml in volume. The visible blood loss post-operation for 186 patients was an average of 89.4 ± 4.0 ml; the hidden blood loss was an average of 277.2 ± 7.6 ml. There were significant differences in the hidden blood loss, the post-operative visible blood loss and the total blood loss between genders in Group PFNA-II (*p* < 0.01). The mean follow-up period was 39 months (range, 37–42 months) for two groups.

**Table 4:** The hidden and visible blood loss of PFNA-II and DHS in the treatment of inter-trochanter fracture (x ± s)

	Number	Operation time (min)	Intraoperation bleed (ml)	Postoperation drainage (ml)	Hidden blood loss (ml)	Visible blood loss (ml)	Total blood loss (ml)
PFNA-II^a^							
Male	81	34.1 ± 1.2	33.0 ± 1.8	53.6 ± 2.1	272.9 ± 6.9	86.6 ± 2.6	359.5 ± 7.0
Female	105	36.5 ± 0.3*	36.0 ± 2.2*	55.6 ± 2.4*	280.5 ± 6.3*	91.6 ± 3.5*	372.1 ± 7.3*
Total	186	35.5 ± 1.4	34.7 ± 2.5	54.7 ± 2.5	277.2 ± 7.6	89.4 ± 4.0	366.6 ± 9.5
DHS^b^							
Male	80	49.9 ± 0.6	100.0 ± 4.3	77.6 ± 3.9	133.0 ± 7.0	177.6 ± 5.6	310.5 ± 9.2
Female	97	52.1 ± 1.1*	103.7 ± 8.3*	79.7 ± 5.1*	144.6 ± 8.1*	183.5 ± 10.3*	328.1 ± 14.2*
Total	177	51.1 ± 1.4^#^	102.0 ± 7.0 ^#^	78.8 ± 4.7^#^	139.3 ± 9.6^#^	180.8 ± 9.0^#^	320.1 ± 15.0^#^

*PFNA-II* Asia proximal femoral nail anti-rotation; *DHS* Dynamic hip screw. *p < 0.01, the male patients vs. the female patients in the same group; ^#^ *p* < 0.01 total patients in Group PFNA-II vs. total patients in Group DHS. Visible blood loss = Intra operation bleed + Post operation drainage. ^a^Smith & Nephew, Memphis, Tennessee; ^b^Synthes, West Chester, PA, USA

In Group DHS, the mean operation time was 51.1 ± 1.4 min. The intra-operation mean blood loss was 102.0 ± 7.0 ml, with no intra-operative blood transfusion. Due to a significant decrease in blood red protein (<8 g/L) on the 2nd-4th postoperative days, 34 patients were provided with blood transfusions, which averaged 400 ± 10 ml in volume. The average visible blood loss and hidden blood loss post-operation for 177 patients were 180.8 ± 9.0 ml and 139.3 ± 9.6 ml, respectively. There were significant differences in the hidden blood loss, the post-operative visible blood loss and the total blood loss between genders in Group DHS (*p* < 0.01).

The operation time was clearly shorter in Group PFNA-II than in Group DHS. The intra-operative blood loss and the post-operative visible blood loss in Group DHS were significantly larger than those in Group PFNA-II. Additionally, the hidden blood loss and total post-operative blood loss were significantly larger than those in Group DHS. There were significant differences between the two groups in terms of the operation time and the blood loss (*p* < 0.01).

## Discussion

Intertrochanteric fractures are associated with significant intra- and post-operative blood loss. When analyzing total blood loss during orthopaedic surgery, the standard is to measure intra-operative bleeding plus postoperative drainage, which ignores hidden blood loss that has been found to be significant in the field of surgery [25]. Additionally, hidden blood loss is also often ignored in clinical practice, due to simpler surgical procedures, shorter operation time and less intra-operative visible blood loss. Therefore, it is easy to aggravate the medical comorbidities or promote the occurrence of other complications.

In elderly high-risk patients, intertrochanteric fractures are more often accompanied by a variety of other diseases. The risk of surgery mainly comes from the medical comorbidities of patients. Hence, the perioperative assessment, the monitoring of vital signs and the necessary measures will be especially important.

Our results are in accordance with the aforementioned studies. In the two groups of patients accepting PFNA-II or DHS treatment, the gender ratio, fracture typing and distribution of complications were consistent and comparable. However, there was a difference in the mean age between men and women in each group, with the mean age of women being significantly higher than that of men. Although the difference does not affect the contrast between the two groups, it may be the reason for the index differences between men and women within each group. Some post-operative patients in both groups presented the symptoms of anaemia, such as feeble muscles, mental fatigue, loss of appetite, haemoglobin decline, etc. According to perioperative statistical data analysis, we discovered that the intra-operative blood loss and the post-operative visible blood loss were less than the true total amount of blood loss. Therefore, it was speculated that a large amount of hidden blood loss exists. This study showed that the mean hidden blood loss in patients undergoing PFNA-II treatment was 277.2 ± 7.6 ml, nearly 75 % of the total amount of blood loss, whereas the mean volume of hidden blood loss in patients treated with DHS was 139.3 ± 9.6 ml, approximately 44 % of the total amount of blood loss. We also found that no matter the PFNA-II group or DHS group, there were significant differences in the comparison of the post-operative visible blood loss, the hidden blood loss and the total blood loss between men and women. In an analysis of the reasons, there may be some differences in the age of men and women within each group. Although the total number of male patients is relatively close to that of female patients, the female patient age was significantly higher, which may be related to the longer life expectancy of females compared to males. That might make the female compensatory ability worse than that of the male [26].

The causes and mechanism of hidden blood loss are not yet clear. The intertrochanteric fracture belongs to the fractures of the metaphysis, which are rich in blood supply. When the intramedullary needle is fixed, the process of expanding the medullary cavity could lead to internal bleeding. In addition, the intramedullary cavities and intramuscular gaps also provide storage cavities for hidden blood loss. Foss, N.B. et al. [28] hypothesized that hidden blood loss could originate from post-operative haemorrhage, the gastrointestinal tract and the initial trauma. Millar, Neal L. et al. [29] suspected intramedullary penetration was associated with hidden blood loss. Jung et al. [9] argued that the main reason for hidden blood loss is the compression of the incision resulting from perioperative blood pouring into the tissue compartments that are not involved in the systemic circulation, resulting in a further reduction of haemoglobin levels, which may be related to an abnormal capillary bed opening caused by free fatty acids, intraoperative intramedullary fat, bone cement and bone debris entering the blood circulation [30,31]. However, at present, there is not yet sufficient evidence to support this inference. Therefore, it is clear that we should put great emphasis on the analysis of hidden blood loss, individualized estimation of total perioperative blood loss, perioperative monitoring, attention to changes in vital signs and urine, control of blood glucose and blood pressure, supplementation of blood levels, prevention of deep-vein thrombosis, and active functional training of the affected limb.

In the present study, the clinical blood loss of patients accepting the treatment of DHS or PFNA-II was analyzed. The results showed that in Group PFNA-II, there was less intra-operative blood loss and more post-operative hidden blood loss. The intra-operative blood loss in Group PFNA-II was less than that in Group DHS. The post-operative hidden blood loss in Group DHS was significantly less than that in Group PFNA-II. According to the data analysis results and clinical experience, PFNA-II is an intramedullary fixation, whose damage to the intramedullary blood supply is of a greater extent compared to DHS. Therefore, the post-operative hidden blood loss of patients receiving treatment with PFNA-II was significantly larger than that of the DHS group. In this study, a post-operative blood losses resulting from two commonly used clinical surgical treatment methods were compared. However, we cannot conclude that the difference in the amount of visible blood loss and hidden blood loss was necessarily caused by the difference in the two kinds of surgical methods, as there was still the existence of hidden bleeding in patients receiving non-surgical treatment of intertrochanteric fractures. Thus, if the reasons could be analzsed to exclude this factor, we could draw conclusions on visible and hidden blood loss resulting from surgical factors. Because clinical intertrochanteric fractures have mostly been treated with surgery in recent years, it is more difficult to obtain the complete data of patients with non-surgical treatment to compare the amount of the hidden blood loss of patients with non-surgical treatment with that of patients with surgical treatment. In addition, because the Group DHS patient cases were mostly completed before 2015, the distribution of the samples was not uniform, which was a limitation of this study. Additionally, no gold standard has been found to measure perioperative blood loss. As a consequence, the evaluation had to be compared with an established technique rather than against the actual quantity. The assessment of the ‘true’ blood loss also had limitations [27].

## Conclusion

The research results fully demonstrated that much postoperative hidden blood loss exists with PFNA-II and DHS in the treatment of intertrochanteric fractures of elderly high-risk patients, and DHS is more favorable than PFNA-II in terms of post-operative hidden blood loss.

## Abbreviations

APTT, activated partial thromboplastin time; CCD, collodiaphyseal angle; CPM, continuous passive motion; DHS, dynamic hip screw; HB, haemoglobin; Hb, haemoglobin; Hct, haematocrit; HCT, haematocrit; HHS, Harris hip score; INR, International normalized ratio; PBV, provisional back-up value; PFNA-II, Asia proximal femoral nail anti-rotation; PT, prothrombin time; SD, standard deviation
